# Biomonitoring using dried blood spots: Detection of ochratoxin A and its degradation product 2’R‐ochratoxin A in blood from coffee drinkers*

**DOI:** 10.1002/mnfr.201500220

**Published:** 2015-06-22

**Authors:** Benedikt Cramer, Bernd Osteresch, Katherine A. Muñoz, Hartmut Hillmann, Walter Sibrowski, Hans‐Ulrich Humpf

**Affiliations:** ^1^ Institute of Food ChemistryWestfälische Wilhelms‐Universität MünsterMünsterGermany; ^2^ Universität Koblenz‐LandauInstitute for Environmental Sciences, Research Group of Environmental and Soil ChemistryLandau in der PfalzGermany; ^3^ Institut für Transfusionsmedizin und TransplantationsimmunologieUniversitätsklinikum MünsterMünsterGermany

**Keywords:** Coffee, Dried blood spot, Exposure, Mycotoxin, Ochratoxin

## Abstract

**Scope:**

In this study, human exposure to the mycotoxin ochratoxin A (OTA) and its thermal degradation product 2’R‐ochratoxin A (2’R‐OTA, previously named as 14R‐Ochratoxin A [22]) through coffee consumption was assessed. LC‐MS/MS and the dried blood spot (DBS) technique were used for the analysis of blood samples from coffee and noncoffee drinkers (*n* = 50), and food frequency questionnaires were used to document coffee consumption.

**Methods and results:**

For the detection of OTA and 2’R‐OTA in blood, a new sensitive and efficient sample preparation method based on DBS was established and validated. Using this technique 2’R‐OTA was for the first time detected in biological samples. Comparison between coffee drinkers and noncoffee drinkers showed for the first time that 2’R‐OTA was only present in blood from the first group while OTA could be found in both groups in a mean concentration of 0.21 μg/L. 2’R‐OTA mean concentration was 0.11 μg/L with a maximum concentration of 0.414 μg/L. Thus, in average 2’R‐OTA was approx. half the concentration of OTA but in some cases even exceeded OTA levels. No correlation between the amounts of coffee consumption and OTA or 2’R‐OTA levels was observed.

**Conclusion:**

The results of this study revealed for the first time a high exposure of coffee consumers to 2’R‐OTA, a compound formed from OTA during coffee roasting. Since little information is available regarding toxicity and possible carcinogenicity of this compound, further OTA monitoring in blood including 2’R‐OTA is advisable.

**Abbreviations**
2’R‐OTA: 2’R‐ochratoxin A
DBS: dried blood spot
FFQ: food frequency questionnaire
IAC: immuno affinity columns
MRM: multiple reaction monitoring
OTA: ochratoxin A

## 1 Introduction

**Figure 1:**
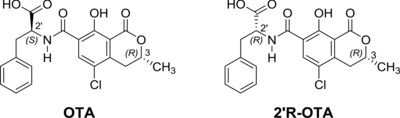
Chemical structures of ochratoxin A (OTA) and 2’R‐ochratoxin A (2’R‐OTA).

Ochratoxin A (*N*‐[(3R)‐(5‐chloro‐8‐hydroxy‐3‐methyl‐1‐oxo‐7‐isochromanyl)carbonyl]‐L‐phenylalanine, OTA, Fig. 1) is a mycotoxin produced by fungi of the genera *Aspergillus* and *Penicillium* 1, 2. It can be found in a large variety of commodities from different climate zones. In northern Europe, especially *Penicillium verrucosum* is responsible for OTA contamination of cereals such as wheat, corn and barley 3, while *Penicillium nordicum* is described as a common OTA producer in meat products such as salami or ham and cheese 3, 4. In warmer climate zones *Aspergillus westerdijkiae, Aspergillus carbonarius*, and *Aspergillus ochraceus* could be identified as potent OTA producers in coffee 5, cocoa 6, grape juice, dried wine fruits 7, spices, and other dried or fermented foods 8, 9.

OTA has been shown to be nephrotoxic, genotoxic, teratogenic, and immunosuppressive in animals 10. Furthermore, OTA has been classified as a possible human carcinogen (group 2B) by the International Agency for Research on Cancer (IARC) due to its nephrocarcinogenicity in animals 11. OTA is able to form DNA adducts after metabolic dehalogenation 12, 13, 14. The role of these adducts on the carcinogenic effect of OTA is under discussion and needs to be further investigated 15, 16, 17. Several studies discussed major food sources responsible for OTA exposure based on the results from the analysis of food samples as well as average consumption data 18, 19, 20. For Europe, cereals, coffee, beer, wine, cocoa, dried fruits, meat, and spices could be identified as the most important sources of OTA 19.

Based on these data, coffee is responsible for approx. 10% of the OTA intake in Germany. Considering that OTA levels are in average lowered by 80–90% during coffee roasting and a binding to coffee beans has been described, OTA exposure due to coffee might even be higher 21. Furthermore, 2’R‐ochratoxin A (*N*‐[(3R)‐(5‐chloro‐8‐hydroxy‐3‐methyl‐1‐oxo‐7‐isochromanyl)carbonyl]‐D‐phenylalanine, 2’R‐ochratoxin A (2’R‐OTA), previously named as 14R‐ochratoxin A, Fig. 1) has been reported as an OTA degradation product with levels in roasted coffee up to 26% in comparison to the OTA concentration 22. Further degradation products of OTA are 2’‐decarboxy ochratoxin A and ochratoxin A amide, of which the latter one has not yet been found in coffee 22, 23. So far, only cytotoxicity assays using human hepatocellular carcinoma (Hep G2) and immortalized human kidney epithelial cells have been performed with these degradation products: 2’R‐OTA is cytotoxic and induces apoptosis in nanomolar or low micromolar concentrations however it is by a factor of 10 less cytotoxic compared to OTA 22, 24. For the other two compounds no or much lower cytotoxicity is reported 22, 23.

Besides the detection in food samples, several studies report the occurrence of OTA in biological samples such as blood and urine. The routine method for the analysis of OTA in these matrices is based on liquid/liquid extraction with chloroform followed by direct analysis, the use of immunoaffinity columns or dispersive SPE clean‐up 25, 26, 27. The respective analytical procedure is usually laborious, requires expensive immunoaffinity columns or is based on halogenated solvents for extraction.

In recent years the use of dried blood spots (DBS) has become popular for the sample collection of human whole blood. Dried blood spots were first introduced by Ivar Christian Bang for the determination of blood sugar levels in 1913 28. This sample technique achieved its breakthrough as a cheap‐screening method for phenylketonuria in the 1960s 29. Samples can be collected on paper cards by pricking toes, heels, ears, or fingers with lancets followed by punching of defined discs with sample material out of the dried cards 30. Alternatively, blood can be spotted with a known volume from an ampoule after venous blood taking. After drying the fresh blood, either the entire DBS is cut out or a disc of defined size is punched out and used for further preparation steps 31. For many analytes DBS protects against humidity, sunlight, high temperatures and can be stored for long time, providing accurate results even after years 32, 33, 34. For analysis, the dried blood samples are extracted with a selective solvent and the resulting extract is analyzed by HPLC‐MS/MS or other sensitive analytical techniques without any further clean‐up 35. In the present work, the DBS technique is applied for the first time for the analysis of OTA in blood samples. Additionally, it is the first approach to detect the thermal degradation product 2’R‐OTA in blood samples from coffee and noncoffee drinkers.

## 2 Materials and methods

### 2.1 Chemicals and reagents

Water for HPLC was purified with a MilliQ Gradient A 10 system (Millipore, Schwalbach, Germany). The reference standards were prepared as described elsewhere 22. Briefly OTA was isolated and purified from fungal cultures. Isotope labeled *d_5_*‐ochratoxin A (*d_5_*‐OTA) was produced by growing *P. verrucosum* on *d_5_*‐L‐phenylalanine enriched growth medium. 2’R‐OTA and *d_5_*‐2’R‐ochratoxin A (*d_5_*‐2’R‐OTA) were prepared by thermal isomerization of OTA and *d_5_*‐OTA, respectively. For all compounds, stock solutions of 1 μg/mL in acetonitrile were prepared.

A working solution was prepared at 20‐fold concentration of the highest calibration point. Blood collection tubes were Monovette^®^ EDTA KE/7.5mL from Sarstedt (Nümbrecht, Germany). Whatman 903 protein saver cards™ for sample collection and preparation were acquired from Sigma‐Aldrich (Taufkirchen, Germany). All other chemicals were purchased either from VWR (Darmstadt, Germany) or Sigma‐Aldrich (Taufkirchen, Germany) and used without further purification.

### 2.2 Blood samples

All participants were informed about scope and aim of this study and gave written consent about their participation. Samples and food frequency questionnaires (FFQ) were number encoded to ensure the anonymity of participants. The design of this study was approved by the research ethical committee of the University Hospital Münster, Germany (file reference: 2014‐187‐f‐S). Participants were 29 men and 21 women between the age of 18 and >60 years. Among the participants, 34 individuals consumed coffee within the past 30 days (coffee drinker) and 16 did not consume coffee within 30 days before sampling (noncoffee drinker).

Venous blood was taken and collected in EDTA‐coated 7.5 mL ampoules. From every blood sample, 100 μL‐spots were placed on DBS cards. The blood spots were allowed to dry at room temperature overnight and cards stored at 4°C in the dark. For analysis, the 100 μL spots were cut out and extracted for 1 h under sonication with 1 mL extraction solution consisting of water/acetone/acetonitrile (30:35:35 v/v/v) and containing 0.01 ng/mL *d_5_*‐OTA and 0.01 ng/mL *d_5_*‐2’R‐OTA. After extraction the filter paper was removed from the vial and the solvent evaporated at 60°C under reduced pressure. The residues were reconstituted with 100 μL water/methanol/formic acid (60:40:0.1 v/v/v) followed by centrifugation for 10 min at 3000 x *g* and injection of 40 μL of the supernatant into the HPLC‐MS/MS system.

### 2.3 HPLC‐MS/MS analysis

#### 2.3.1 HPLC‐MS/MS system

A QTRAP 6500 MS system (SCIEX, Darmstadt, Germany) coupled to a 1260 Infinity LC system (Agilent, Waldbronn, Germany) was used for the detection in multiple reaction monitoring (MRM) mode. Data acquisition was performed with Analyst 1.6.2 software. The chromatographic separation was carried out on a 150 × 2 mm inner diameter, 5 μm, Nucleodur C18 ISIS column and a 2 × 4 mm guard column of the same material (Macherey‐Nagel, Düren, Germany) at a column temperature of 40°C. Flow rate was 0.3 mL/min and solvent A was methanol and solvent B water, both containing 0.1% formic acid. The binary gradient was programmed as follows: 0 min, 60% A; 3 min, 60% A; 6 min, 100% A; 10 min, 100% A; 10.1 min, 60% A; 14 min, 60% A. The mass spectrometer was operated in the positive mode. For the electrospray ionization a voltage of +5500 V was used, the potentials for fragmentation were set as follows: Declustering potential +35 V; entrance potential +8 V; collision cell exit potential +11 V; and collision energy potential +31 V. The transition reactions were monitored with duration of 100 ms each. The selected MRM transitions for OTA and 2’R‐OTA were *m/z* 404 → *m/z* 239 as quantifier and *m/z* 404 → *m/z* 102 as qualifier. For *d_5_*‐OTA and *d_5_*‐2’R‐OTA *m/z* 409 → *m/z* 239 and 409 → *m/z* 102 were used as quantifier and qualifier, respectively.

#### 2.3.2 Calibration

A seven‐point calibration curve for OTA and 2’R‐OTA was set in concentration range from 0.005 to 1.0 ng/mL. Each calibration point contained 0.1 ng/mL of both isotope labeled standards *d_5_*‐OTA and *d_5_*‐2’R‐OTA. The calibration solutions were stable over 24 h at 4°C. Reproducibility was determined by means of a quality control sample that was taken from the cohort, containing both OTA and 2’R‐OTA. It was analyzed with every sample batch and measurement was accepted if the determined concentration ranged between 80 and 120% of the mean value. No decrease of the OTA or 2’R‐OTA concentrations was observed in the control sample, showing that both analytes are stable for at least four weeks when stored at 4°C on the DBS cards. The recovery rates were determined for four different analyte concentrations as described below. The LOQ and the LOD were determined in spiked samples via the S/N with thresholds of 1–3 and 1–10, respectively.

#### 2.3.3 Recovery rates

As no human blood sample was available without detectable concentrations of OTA, the sample with the lowest OTA concentration of 0.096 ± 0.003 ng/mL and no detectable 2’R‐OTA was used for the determination of the recovery rates. Determination of the recovery rate was performed in triplicate at four different spiking levels. For each concentration level 1 mL of blood was spiked with 5 to 20 μL of a solution containing OTA and 2’R‐OTA at concentrations of either 10 ng/mL or 1 ng/mL. The resulting concentrations were 0.05, 0.1, 0.25, and 1 ng/mL for both, OTA and 2’R‐OTA. Subsequently the samples were analyzed as described above.

### 2.4 Food frequency questionnaire

The design of the FFQ that was filled out by all participants of the study was based on Gerding et al. 36 and covered long and short‐term nutritional habits (recall of past 30 days and 24 h recall). For this study, the consumption of coffee and soluble coffee was requested in the range from 0 to 0.7 L/day and >0.7 L/day. The FFQ was generated and automatically processed with Evasys V5.1 software. A statistical analysis was carried out to identify significant correlations between coffee consumption and OTA/2’R‐OTA concentrations found in blood. Samples were grouped according to the consumption frequencies stated by the participants. For comparison, independent sample *t*‐test was applied.

## 3 Results

### 3.1 Method development

**Figure 2:**
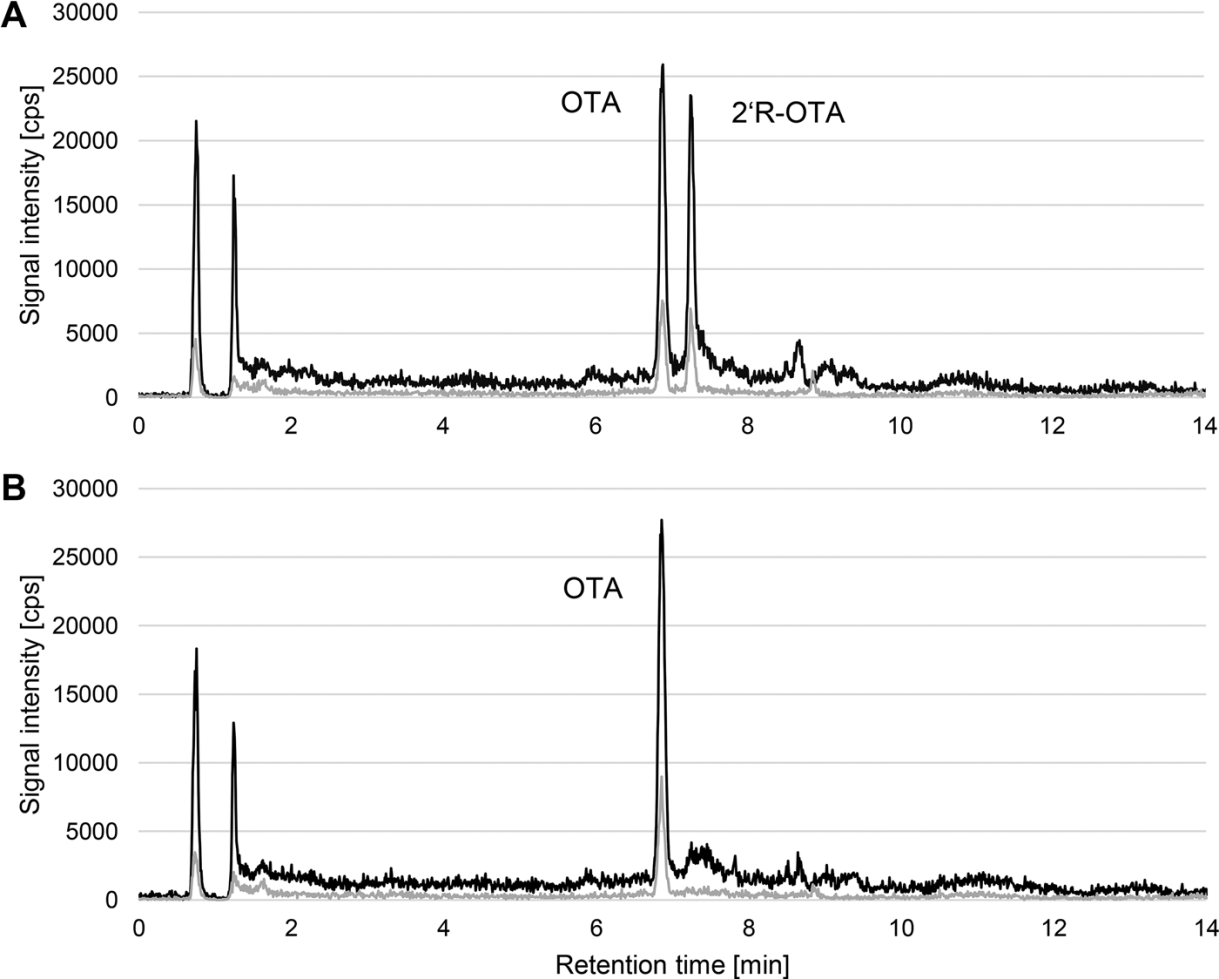
HPLC‐MS/MS chromatogram of a DBS extract from a coffee drinker containing 0.264 ng/mL OTA and 0.273 ng/mL 2’R‐OTA (A) and a noncoffee drinker containing 0.284 ng/mL OTA and no detectable 2’R‐OTA (B). Gray: *m/z* 404.0 → *m/z* 239.0, black: *m/z* 404.0 → *m/z* 102.0.

HPLC‐MS/MS analysis of OTA was performed in positive ion mode with electrospray ionization under standard conditions 37. Particular attention was paid to the column selection and the solvent gradient in order to achieve a baseline separation of OTA and 2’R‐OTA, as both compound show identical fragmentation patterns, making a mass spectrometric differentiation impossible. For sample preparation 100 μL of blood samples were applied on a DBS card, dried, cut out and extracted using a mixture of 35% acetonitrile 35% acetone 30% water containing *d_5_*‐OTA and *d_5_*‐2’R‐OTA as internal standards. The extract was evaporated to dryness, and after reconstitution and centrifugation a clear, nearly colourless extract was obtained. For HPLC‐MS/MS analysis of the extract, MRM transitions *m/z* 404.0 → *m/z* 239.0 and *m/z* 404.0 → *m/z* 102.0 yielded the best sensitivity (Fig. 2). Based on the S/N ratios of 3 and 10 for the least sensitive MRM transition *m/z* 404.0 → *m/z* 239.0, without matrix, LOD and LOQ were 0.005 and 0.013 ng/mL for both, OTA and 2’R‐OTA, respectively. As no sample without OTA was available, the LOD and LOQ in matrix could only be determined for 2’R‐OTA and were 0.005 and 0.021 ng/mL, respectively, which is far below the lowest OTA concentration measured. Both analytes showed linear calibration curves in the respective calibration range with a coefficient of determination (*R* ^2^) between 0.997 and 0.999. The recovery rates were determined in triplicate and ranged between 101–105% for OTA and 99–105 % for 2’R‐OTA at blood concentrations of 0.05, 0.10, 0.25, and 1.0 ng/mL, respectively. Reproducibility was investigated by the analysis of a control sample over a period of five days and was 7.3% for OTA and 7.5% for 2’R‐OTA.

### 3.2 Biomonitoring in blood samples from coffee drinker and noncoffee drinker

Fifty blood samples from healthy adults from the area of Münster, Germany were analyzed using the new DBS‐based method. OTA could be detected in all blood samples within a concentration range of 0.071–0.383 ng/mL and in a mean concentration of 0.21 ± 0.066 ng/mL. As shown in Table 1, no statistical difference between the OTA levels in blood of coffee and noncoffee drinkers could be determined. Also between male and female no significant difference was found.

**Table 1:** Levels of OTA and 2’R‐OTA (ng/mL) in blood according to gender and coffee consumption

	*n*	Mean ± SD	Median	Range
OTA (ng/mL)				
Female	21	0.19 ± 0.056	0.19	0.071–0.302
Male	29	0.23 ± 0.069	0.22	0.113–0.383
All	50	0.21 ± 0.066	0.21	0.071–0.383
Coffee drinker	34	0.21 ± 0.067	0.21	0.071–0.383
Noncoffee drinker	16	0.21 ± 0.065	0.21	0.113–0.325
2’R‐OTA (ng/mL) (coffee drinker only)				
Male	19	0.13 ± 0.110	0.11	0.023–0.414
Female	15	0.09 ± 0.066	0.06	0.021–0.271
All	34	0.11 ± 0.093	0.07	0.021–0.414

**Figure 3:**
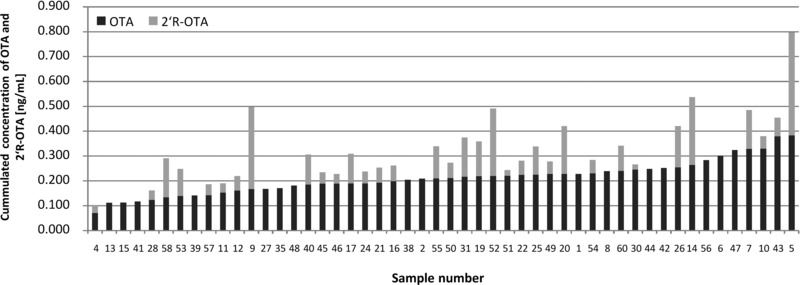
Distribution of OTA (black) and 2’R‐OTA (gray) in DBS samples.

**Figure 4:**
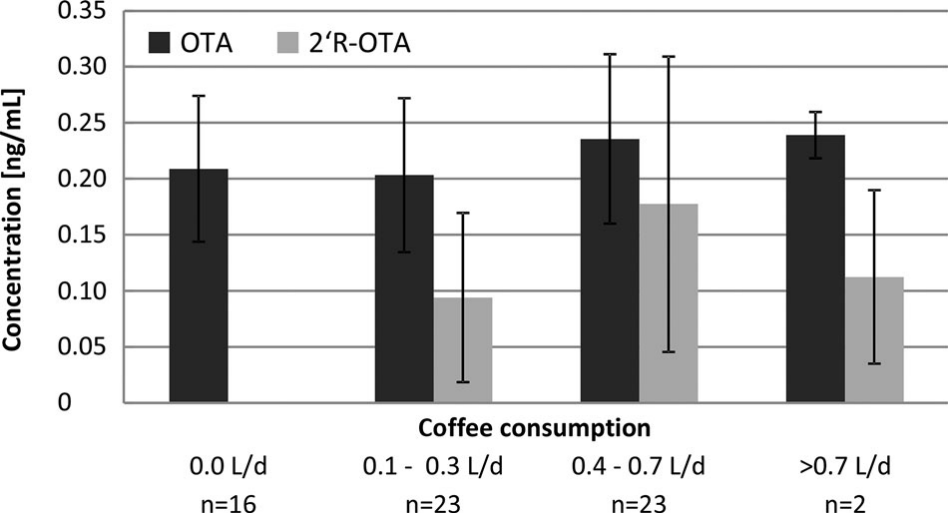
OTA (black) and 2’R‐OTA (gray) concentrations [ng/mL] related to coffee consumption.

2’R‐OTA could be detected in all blood samples from coffee drinkers in a range between 0.021 and 0.414 ng/mL while no 2’R‐OTA was found in samples from the noncoffee drinker group. The mean concentration of 0.11 ± 0.093 ng/mL for 2’R‐OTA in the coffee drinker group is approx. half of that of OTA. However, as shown in Fig. 3, for individual samples strong variations of the ratio of OTA to 2’R‐OTA can be observed. With some samples such as numbers 5, 9, and 52 having 2’R‐OTA concentrations even exceeding those of OTA. In the FFQ, the average amount of coffee beverage consumed per day during the last month was requested. Comparison of the OTA and 2’R‐OTA shown in Fig. 4 revealed no statistical difference between the groups except for 2’R‐OTA, which was not found in the noncoffee drinker group.

## 4 Discussion

In this study, an innovative, simple, and efficient method for the detection of OTA and 2’R‐OTA in human blood samples was developed, based on the dried blood spot analysis of pharmacological active compounds 38. Compared to liquid‐liquid extraction, SPE, or immuno affinity columns (IAC), which are currently used for the analysis of OTA in blood, the new method provides essential benefits. This clean up method can be performed simultaneously for 40 or more samples and only little waste is produced. It is fast, omits halogenated solvents and does not require expensive clean‐up cartridges. Besides OTA the simultaneous analysis of 2’R‐OTA can be performed that is not bound by most IAC columns 22. Additionally, the use of the DBS‐technique gives a possibility for simple storage and shipping of blood sample material, allowing an easy transport of samples from the blood donor to the analytical facility. Additionally, the LOD and LOQ obtained with the new method are below or equal to those found in literature while sample consumption is minimized 26, 27, 39. Recoveries around 100% and a good repeatability confirm the suitability of the DBS technique for OTA biomonitoring studies.

Application of the DBS method to 50 samples from healthy adults showed a close distribution of most OTA concentrations around the mean value with only few higher concentrations as shown in Fig. 3. The lack of “extreme” OTA blood levels can be explained by the fact that OTA blood levels are primarily a long‐term marker for OTA exposure and not so much influenced by a single high dose 40, 41. On the other hand, it is rather unlikely that the participants consumed regularly highly contaminated food as OTA regulation is comparably strict in Germany and Europe. Thus the determined mean concentrations given in Table 1 are in good agreement with previously published concentrations of 0.28 and 0.23 ng/mL for adults in Germany 39, 42. Comparison of the OTA concentrations in the blood samples from coffee and noncoffee drinkers showed no significant (*p* > 0.05) difference in the mean concentration as shown in Fig. 4.

In Germany cereals (0.65 ng/kg bw/day), coffee (0.14 ng/kg bw/day), beer (0.05 ng/kg bw/day), cocoa (0.07 ng/kg bw/day), and meat (0.06 ng/kg bw/day) are the most important sources for OTA exposure 19. Thus based on the calculated impact of coffee consumption on the overall intake of OTA as well as the concentrations of 2’R‐OTA found in roasted coffee, only trace amounts of 2’R‐OTA were expected to be found in blood. Instead, among the samples analyzed, some contained even higher concentrations of 2’R‐OTA than OTA. Possible explanations for this effect could be that more OTA and 2’R‐OTA are present in coffee than detected with the current methods. This possible masking could be due to matrix binding and release of OTA and 2’R‐OTA in the gastrointestinal tract 21. However, in this case we would expect that also higher levels of OTA can be found in the blood from coffee drinkers, which is not the case. Alternatively, a reduced excretion or an enhanced resorption of 2’R‐OTA compared to OTA could be a reason for this effect. Compared to OTA that already has a biological half‐life‐time of approx. 35 days 17, this would indicate a very high persistence of 2’R‐OTA in the body. Further sources of 2’R‐OTA were not identified in the study, but can also not be excluded.

Strong interindividual differences in the 2’R‐OTA levels were observed. Explanations for this effect could be related to coffee processing, coffee quality or differences in the metabolism of 2’R‐OTA but need to be further investigated.

## 5 Summary and conclusion

A DBS‐based sample preparation technique for the detection of OTA and its thermal degradation product 2’R‐OTA in blood was developed and validated. Due to the use of DBS, the new method simplifies sample treatment and allows the storage of samples for OTA analysis as well as shipping with almost no restriction compared to fresh blood samples. Furthermore, sample clean‐up is simplified and accelerated compared to published procedures.

The new technique was applied for the analysis of blood samples from coffee consumers and noncoffee consumers in Germany. Besides OTA its thermal degradation product 2’R‐OTA was analyzed for the first time in physiological samples. While no significant differences in the OTA levels between both groups were observed, 2’R‐OTA was found in all blood from coffee drinkers in levels up to 0.414 ng/mL, exceeding even the highest OTA concentration of 0.383 ng/mL detected in the study. In average, 2’R‐OTA concentration was 0.11 ng/mL, which is half of the mean OTA concentration of 0.21 ng/mL, and thus much higher than expected, considering the levels of 2’R‐OTA typically found in roasted coffee and the limited impact of roasted coffee on the overall OTA exposure. As 2’R‐OTA is cytotoxic in cell culture in the nanomolar or low micromolar range (although less cytotoxic compared to OTA) and no additional information about the toxicity and toxicokinetics of this compound is available, further research is needed to understand the relevance/contribution of this compound for human exposure to mycotoxins. OTA biomonitoring studies that include 2’R‐OTA are advisable.
